# Validation of a computerized driving simulator test of cognitive abilities for fitness-to-drive assessments

**DOI:** 10.3389/fpsyg.2023.1294965

**Published:** 2024-01-08

**Authors:** Max Bremberg Gårdinger, Robert Johansson, Björn Lidestam, Helena Selander

**Affiliations:** ^1^Department of Psychology, Stockholm University, Stockholm, Sweden; ^2^Swedish National Transport Research Institute, Linköping, Sweden; ^3^Swedish National Transport Research Institute, Gothenburg, Sweden; ^4^Department of Clinical Neuroscience, Institute of Neuroscience and Physiology, Sahlgrenska Academy, University of Gothenburg, Gothenburg, Sweden

**Keywords:** cognition, driving assessment, simulator, off-road test, attention, fitness to drive

## Abstract

**Background:**

Driving requires a series of cognitive abilities, many of which are affected by age and medical conditions. The psychosocial importance of continued driving ushers the need for valid measurements in fitness-to-drive assessments. A driving simulator test could prove useful in these assessments, having greater face validity than other off-road tests and being more cost-effective and safer than ordinary on-road testing. The aim of this study was to validate a driving simulator test for assessment of cognitive ability in fitness-to-drive assessments.

**Methods:**

The study included 67 healthy participants. Internal consistency of the simulator subtests was estimated. A correlation analysis between results on the simulator and the cognitive tests Trail Making Test (TMT) A and B and the Useful field of View test (UFOV) and multiple regression analysis were conducted. Finally, a comparison of results between age groups (>65 years) and (<65 years) was done.

**Results:**

Results showed good internal consistency. Significant and moderate correlations were found for all reaction time in the simulator’s subtests and UFOV 3, and all but two with TMT A. Lane positioning in the simulator showed significant and low to moderate correlations with UFOV 3 in all subtests. Reaction time and Double reaction time on subtest 3 were significantly correlated with UFOV 2 and UFOV 3 and TMT A, respectively. Test on Centerline (position) in subtest 3 as dependent variable was significantly correlated with UFOV 3. Significant means differences and large effect sizes between the age groups were found for all reaction time and lane positioning tests.

**Conclusion:**

The findings of concurrent validity, especially with TMT A and UFOV 3 and its sensitivity for age-related differences, indicate potential for the simulator to be used as a complement in fitness-to-drive assessments. However, a clinical study is necessary to further examine its usefulness for patients with cognitive deficits.

## 1 Introduction

For many individuals, a driver’s license is a necessity to be able to get to and from work, attend to everyday commitments and maintain social contacts. For elderly drivers, continued driving also contributes to, and constitutes, an important factor for the individual’s self-confidence, wellbeing and social network (Adler and Rottunda, 2006). Further, older drivers who have their driver’s license revoked suffer from decreased life satisfaction, loss of personal identity and greater health problems, such as depression (Stutts and Wilkins, 2003). Hence, for many older drivers, the psychosocial significance of continued holding of a driver’s license is not to be neglected.

Driving a car is in many ways an automized task, however, being able to respond to different traffic situations and occurrences requires sufficient and adequate functioning of visual, motor and cognitive abilities (Eby et al., 1998; Lundqvist, 2001). From a cognitive perspective, these include attention and visual processing, but also memory and various executive functions (Anstey et al., 2005; Mathias and Lucas, 2009). Many of these cognitive abilities are prone to decline with age or by medical conditions such as stroke or Alzheimer’s disease (Classen et al., 2013).

In Sweden, and many other industrialized countries, people over the age of 65 is the fastest growing age group (McGwin et al., 2000; Statistics Sweden, 2022). In 2022 there were around 2.2 million people over the age of 65 in Sweden. According to authorities, 87% of individuals in the age of 65–79 and 80% of individuals over the age of 80 possess a driver’s license (Transport analysis, 2022). There is no statistics on how many of these individuals who are active drivers, however projections are that there will be a continuous increase in the number of people over the age of 65 who will continue to drive higher up in ages (McGwin and Brown, 1999; Shanmugaratnam et al., 2010).

When looking at the number of licensed drivers in general, older drivers are involved in fewer crashes and traffic incidents, however, they do have a higher crash rate per kilometer driven, as compared to middle-aged drivers (Eby et al., 1998; McGwin and Brown, 1999). When examining characteristics of traffic incidents among young, middle-aged and older drivers, McGwin and Brown (1999) found that older drivers were more likely to have accidents in environments that requires good attention, i.e., at intersections, in lane changes, to fail to yield way, to fail to see objects and to fail to heed stops signs, as compared to the other two age groups. Younger drivers were more likely to be involved in accidents due to higher risk taking, such as driving at high speed, in bad weather conditions and with driver fatigue, when compared to older drivers (McGwin and Brown, 1999). This indicates that although older drivers are less risk-taking, they are at greater risk of crashes due to decline in attention and perceptual skills. Further, several neurological conditions, such as Alzheimer’s disease and stroke, have been shown to affect safe driving, which has been shown both in poorer driving performance, on both on- and off-road tests, and as higher crash rates (Anstey et al., 2005; Mathias and Lucas, 2009).

However, there are large individual differences in driving capacity among older drivers and many have a great driving experience and compensate for possible decline in cognition or physical ability, hence age alone should not be a criterion for evaluation of driving capacity (Owsley et al., 1998; Shanmugaratnam et al., 2010). Further, a diagnosis alone does not necessarily mean that the individual is not fit to drive. The individual might still have the cognitive or physical abilities necessary for driving intact, or is able to compensate for his or her decline in important abilities (Anderson et al., 2005). Instead, focus should be on determining and measuring those abilities relevant for safe driving (Mathias and Lucas, 2009).

In the literature, the act of driving is described in many ways, with somewhat different interpretations. *Driving capacity*, *driving behavior*, and *fitness to drive* are terminological concepts being described and used in this study (Lee et al., 2003; Anstey et al., 2005; Marshall et al., 2007; Shanmugaratnam et al., 2010). *Driving capacity*, sometimes referred to as *driving ability*, describes the actual cognitive, perceptual and motoric capacity of an individual to operate a vehicle in a safe way (Lundqvist, 2001). *Driving behavior* refers to the way the individual acts while driving. Hence, this concept is viewed as to include all the abilities needed for safe driving. This concept is sometimes also referred to as *driving performance* in the literature (Shanmugaratnam et al., 2010; Devlin et al., 2012). However, the term *driving performance* is often also used to describe the outcome on an on- or off-road test (Marshall et al., 2007). Thus, *driving behavior* is the term used in this study, regarding the way in which an individual behaves and acts in a driving situation. When the term *performance* is used, it refers to test outcome. *Fitness to drive* refers to the medical demands an individual with some type of medical condition needs to meet in order to be considered as fit to drive. In Sweden, these demands are stated in legislature and include both cognitive and physical abilities (Swedish Transport Agency, 2010:125) and are closely related to the skills included in the concept of *driving capacity.*

Several cognitive abilities have been found to predict crash risk among drivers (Anderson et al., 2005; Mathias and Lucas, 2009; Shanmugaratnam et al., 2010). Deficits in cognitive abilities, such as attention, processing speed, perception, executive functions and memory, are likely to account for a significant proportion of accidents involving older drivers (Anstey et al., 2005; Shanmugaratnam et al., 2010; Selander et al., 2020).

Anderson et al. (2005) developed a model for understanding how cognitive abilities are involved in driving and driving behavior. The model illustrates the link between a driving situation and its driving outcome, regarding safe or unsafe driving. A way of understanding the model is, when presented to an occurring driving situation, a sequence of steps requiring a set of cognitive abilities put into action. Step one involves the ability to perceive visual stimuli and depends on cognitive abilities such as attention, visuo-spatial abilities and perception. Step two involves the ability to interpret the situation based on earlier experience in combination with the current situation at hand, and demands processing speed and attentional abilities. The third and final step involves the action being taken in accordance with the current situation, for example braking. In accordance with other studies, the series of steps included in the model requires executive skills, such as mental flexibility and action-planning, attention skills and perceptual skills, but are also mediated by declarative and procedural memories regarding earlier driving situations (Anderson et al., 2005; Devlin et al., 2012).

In previous research, attention, specifically visual attention, has repeatedly shown to be a cognitive ability with high predictive value in discriminating between drivers being considered fit or unfit to drive, respectively, (McKenna, 1998; Mathias and Lucas, 2009). Attention is a cognitive ability consisting of different facets: sustained, divided and selective attention. Divided attention and selective attention are the two facets that have shown the highest predictive value in terms of crash risk, especially in older drivers and individuals with neurological conditions (Eby et al., 1998). Sustained attention refers to the ability to maintain attention on a specific stimulus for a longer period of time. When driving, it is necessary to maintain attention on other road users and road signs for example. Divided attention refers to the ability to process information while simultaneously doing something else while selective attention refers to the ability to filter out visual stimuli to focus on the task at hand (McKenna, 1998; Montes et al., 2016; Wolfe and Lehockey, 2016). However, in order to drive safely, a driver needs to be able to shift attention between different, competing, stimuli while conducting several tasks at the same time (Classen et al., 2013). One example is being able to turn or merge while at the same time keeping track of potential conflicting stimuli, such as pedestrians or traffic signs, depends on the driver’s attention skills (Classen et al., 2013).

Driving is undoubtedly a visual task, and besides the importance of visual acuity, cognitive aspects of perception are necessary, such as visual perception and visuo-spatial abilities (Marshall et al., 2007). Safe driving requires the ability to perceive your surroundings and accurately respond to changing visual stimuli (Moran et al., 2020). Further, visuo-spatial ability refers to the cognitive skill of observing and understanding the visual and spatial relationship between objects. This is of great importance in several driving situations, such as when passing on-coming traffic, especially when there is limited space for error (McKenna, 1998).

Reaction time, that is, the ability to process information in a fast and adequate way, has implications for driving capacity since it is relevant for responding accurately and quickly to various situations (Etienne et al., 2013; Wolfe and Lehockey, 2016). Reaction time is dependent on sensory, cognitive, and motor abilities. Age-related decline in reaction time has been found to be primarily due to slowed information processing, hence primarily of cognitive nature rather than caused by decline in motor or sensory abilities (Eby et al., 1998). Slow processing speed can result in slow and hesitant driving and unexpected maneuvers (Eby et al., 1998). It is also associated with memory decline, especially for short-term memory, since reduced speed of processing affects how quickly the individual can receive information held in short-term memory. This has implications for driving, since information about potential hazards must be responded to quickly (Eby et al., 1998).

The executive functions involve several different aspects of cognitive abilities, such as judgment, planning, mental flexibility and response inhibition, all relevant for both driving behavior and driving capacity (Wolfe and Lehockey, 2016). Executive functions further allow anticipation and adaptation of behavior in accordance with changing environments (Etienne et al., 2013). This is for example necessary when adapting to a less automatic maneuver. Deficits in certain executive functions might limit the driver in making a safe behavioral change by taking into account the potential risks involved and the self-evaluation of own capacities (Daigneault et al., 2002). This is associated with, among other functions, the executive aspect of mental flexibility, which allows for adequately shifting between cognitive tasks (Etienne et al., 2013). Response inhibition refers to the ability to inhibit automatic responses, which in relation to driving has shown importance in terms of suppressing responses when sudden changes occur and call for an alternative action, such as waiting at an intersection (Moran et al., 2020).

Driving capacity has been found to be closely linked to cognitive functioning, all of which is affected naturally with age and when suffering from many medical conditions (Selander et al., 2011). Cognitive tests and the understanding of the cognitive processes involved in driving can provide valuable insights in determining an individual’s driving capacity (Mathias and Lucas, 2009; Selander et al., 2020), hence understanding and assessing these cognitive processes can help identify deficits in driving capacity in a fitness-to-drive assessment. Countries have different regulations regarding fitness-to-drive assessments, but generally these assessments involve a medical evaluation and off-road tests, and to some varying degree on-road testing (Korner-Bitensky et al., 2006; Mathias and Lucas, 2009). In Sweden physicians are legally obliged to report to license authorities if they deem a patient unfit to drive (Swedish Transport Agency, 2010:125). There are several different conditions stated, among these are cognitive disorders or impairments, such as Alzheimer’s disease and stroke (Swedish Transport Agency, 1998:488).

When assessing the level of cognitive function, a neuropsychological assessment is often done through different types of off-road tests, measuring cognitive abilities deemed relevant for driving, and forms the basis for decisions on whether there are cognitive impairments for continued driving (Swedish Transport Agency, 2010:125). An assessment should gather information from several cognitive domains and make a collective assessment, partly through off-road test results, partly through simulated or real driving situations (Samuelsson et al., 2018; Moran et al., 2020).

On-road driving is considered the golden standard regarding assessing driving capacity and driving behavior, since it has high face validity (Mathias and Lucas, 2009; Samuelsson and Wressle, 2021). However, on-road assessments might be impractical, costly and have shown to be stressful to older drivers (Lee et al., 2003). Further, safety considerations must be made to ensure safety of the individual and the public, if allowing a potentially unsafe driver to manage a vehicle in an on-road test (Mathias and Lucas, 2009). It is still considered to be insufficient only to rely on an on-road assessment and fitness-to-drive assessment should be complemented with results on standardized cognitive tests as well (Selander et al., 2020).

Simulator tools have been used for measurement of driving capacity, with varying results (Lee et al., 2003; Anderson et al., 2005; Shanmugaratnam et al., 2010; Samuelsson et al., 2018). Simulator tests have the potential to measure cognitive ability in a standardized manner, whilst in a driving-like situation, and further have an added value in the possibility to observe other aspects of driving behavior (Lee et al., 2003; Samuelsson and Wressle, 2021). As opposed to an on-road assessment, the simulator has the advantage of providing controlled and reproducible situations and can be used for both older drivers in general, but also for individuals with neurological conditions (Etienne et al., 2013). Unlike most other off-road tests being used in clinical practice, such as TMT A and B (Trail Making Test, Tombaugh, 2004) and the UFOV test (Useful Field Of View test, Wood and Owsley, 2014), a simulator tool has high face validity, that is, what the test ocularly appears to measure and whether it *appears* valid to the individual (Blana, 1996). Considering the psychosocial importance and the important consideration needed when assessing driving capacity, the face validity of a test instrument is an important aspect to consider in fitness-to-drive assessments (Kay et al., 2012). Hence, a simulator tool could have important implications in fitness-to-drive assessments by adding higher face validity than other off-road tests yet being more cost efficient than on-road testing (Brown and Ott, 2004).

The subject of older drivers has been researched for decades and with an ageing population, the relevance for further explorations and research is possibly even more relevant than before. A fitness-to-drive assessment needs to evaluate driving capacity in a cost-effective and reliable way. Many neuropsychological tests are aimed at measuring cognitive abilities relevant for driving, however, what relevance these test results have specifically for driving capacity is not always clear (Lundqvist, 2001). The aim of the present study was to conduct an initial validation of a simulator tool for assessment of cognitive aspects of driving capacity, by examining the reliability and validity for the simulator test. The study further aimed at examining age differences in cognitive abilities relevant for driving, in order to investigate the sensitivity to age differences of the simulator tool, and thereby create a first set of norm-based values for future standardized testing. Based on the knowledge of age-related cognitive decline, the hypothesis was that there would be a significant difference between the two groups, with the older age group performing more poorly on the simulator tasks.

## 2 Materials and method

### 2.1 Participants

Participants (a younger age group, 18–65 years old) were recruited through an existing list based on individuals interested in participating in scientific studies at The Swedish National Road and Transport Research Institute (VTI). Participants over the age of 65 were recruited via The Swedish Pensioners’ Association.

Inclusion criteria for participation were possession of a driver’s license for passenger car (type B according to Swedish regulation). The participant also had to be an active driver. However, no limited amount in terms of kilometers per year was set. Exclusion criteria were individuals having suffered from stroke, dementia or had other medical conditions or medication that can affect cognition, for example age-related diabetes. These, and all other descriptive variables, such as age, driving habits and education were self-reported.

A total of *N* = 72 participants aged 19–87 years showed interest in the study. These were contacted by phone or email for an initial screening of exclusion and inclusion criteria and for the participant to get the opportunity to ask questions about the study. During the test situation, two participants reported having suffered from stroke, which had not been reported during screening. In addition, three participants chose to discontinue the study due to motion sickness (so called simulator illness) during testing. After excluding these, 67 participants were included in the analysis, whereof 30 men and 37 women, with a mean age of 53.2 (*SD* = 21.0) years. The sample was split into two age groups in order to test effects of age and age-related differences, see Table 1 for demographic data. The younger age group (<65 years) had 17 men and 27 women; the older age group (≥65 years) had 13 men and 10 women.

**TABLE 1:** Means, standard deviation and range of demographic data for the sample by age groups.

	< 65 years (*n* = 44)			≥65 years (*n* = 23)		
	*M*	*SD*	Range	*M*	*SD*	Range
Age (years)	40.7	13.9	19–59	77.1	5.6	66–87
Education (years)	16.9	3.6	12–26	15.0	2.5	12–22
Driving habits (km/year)	1491	944	50–3000	1104	769	200–4000

### 2.2 Materials

#### 2.2.1 Simulator

**FIGURE 1:**
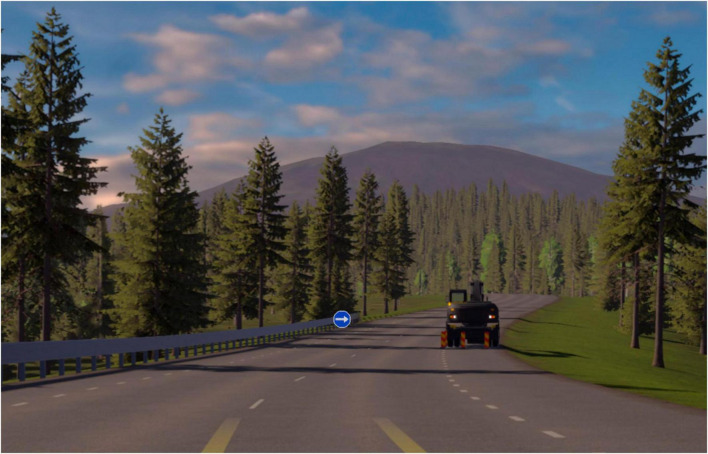
The simulator scenario with an example of a road sign (arrow) and an obstacle. Reprinted with permission from Skillster AB.

The simulator is based on an earlier Dutch simulator, developed to measure attention skills and simultaneous capacity (Brouwer et al., 1991). Both the original simulator and updated versions of the simulator have been used at several clinics in Sweden for fitness-to-drive assessments. The simulator being tested in this study has been further developed in collaboration between VTI and Skillster AB, a Swedish company specialized in constructing simulators for driving education. The simulator is used on a regular PC with an attached 50-inch screen and a steering wheel (Figure 1). The steering wheel is equipped with two paddles (one on the left-hand; one on the right-hand side), used for responding to different stimuli appearing on the screen during the tests.

The driving takes place on a predetermined route, a rural road, which is the same route for all subtests in the simulator program. To minimize the risk of simulator sickness, the route does not include any turnings or crossings, and the participant should therefore only drive straight ahead. Furthermore, along the road there are some obstacles that the participant needs to keep attention to, e.g., road cones for a roadwork, a parked bike, or a parked vehicle. The “car” travels at a constant speed of 80 km/h, which means that the participant does not need to accelerate or brake, also a reason for minimizing simulator sickness. The participant’s task is to steer the “car” straight ahead in the right lane and at the same time maintain good attention and concentration, which is required for safe driving. While the participant is driving, an attention and reaction test is carried out, such that two different types of road signs (arrows) are shown on the right-hand or the left-hand side of the screen. The road signs have the same size throughout the test and stay on screen until the participant presses the paddle, but no longer than 4 s, when the road sign disappears of itself. The road signs appear on the same horizontal line, however, they can appear on six different locations. The driver must react by pressing the respective (correct/incorrect) paddle on the steering wheel as quickly as possible. Further, during subtests 2 and 3, the participant is also asked *not* to react to signs pointing in different directions. This demands response inhibition, which is an executive function found relevant for safe driving (Wolfe and Lehockey, 2016).

The program consists of a driving scenario (approximately 25 min) divided into three sub-tests. Before the simulator runs, the participant receives information on how to perform the tasks and can practice before each subtest. The subtests have an increasing level of difficulty with regard to attentional skills as the test progresses.

1. Subtest 1 consists of a driving situation on a one-way rural road (the same road in all subtests). The participant is asked to steer the car within the right lane while keeping a straight position, avoiding crossing the centerline or the right edge line (offroad). Further, the participant is instructed to avoid any meeting cars (*n* = 3) and obstacles (*n* = 3) occurring during the test. When an obstacle appears, the participant is told not to run into it. During the test, arrows (the road signs), either pointing to the left or to the right, appear on different locations of the screen. The participant is told to press the right paddle on the steering wheel if the arrow is pointing to the right, and the left paddle if the arrow is pointing to the left. Since the arrows can appear both on the left-hand and the right-hand side, it is vital that the participant keep and maintain good attention and notices the direction of the arrow, and not just respond to the side on which the symbol is shown. The subtest lasts around 5 min and consists of 32 stimulus sequences.
2. Subtest 2 has the same basic set-up, with driving on the same road and meeting cars (*n* = 3) and obstacles appearing at random times (*n* = 3). However, this time two arrows are shown simultaneously, on both sides (i.e., left and right) of the screen. The arrows can either be pointing in the same direction (i.e., both arrows to the right, or both arrows to the left), or they could point in different directions. If the arrows point in the same direction, the participant is told to double-click on the corresponding paddle, that is, if both arrows point to the left, the participant should double-click the left paddle; if both point to the right, the participant should double-click the right paddle. If the arrows point in different directions, the participant is told not to press any of the paddles, hence the need to inhibit the impulse of pressing the paddles. This subtest lasts about 5 min and also consists of 32 stimulus sequences.
3. Subtest 3 has also the same set-up, with driving on the same road and meeting cars (*n* = 4) and obstacles appearing at random times (*n* = 4) but the task is a combination of the two earlier subtests. In this test both single and double arrows are shown. When one arrow is shown, the rules of the first subtest are to be followed; and when two arrows are shown, the rules of the second subtest are to be followed. This is the longest subtest and lasts around 8 min and consists of 54 stimulus sequences (28 single arrows; 26 double arrows), hence it is considered to also measure cognitive stamina.

The data generated from the simulator test are; mean values for reaction time, number of error (incorrect responses) or misses (no responses) for the road signs/arrows on the respective subtests. In addition, difficulties with lane position (number of times crossing the centerline or edge line/offroad), and collisions with oncoming traffic and obstacles are also reported. Results on the variables for reaction are in milliseconds. Position–Centerline, Position–Edge line/offroad, and Misses and Incorrect reactions are in number of errors made.

#### 2.2.2 Trail making test A and B

The trail making test (TMT) is a “paper and pencil” test consisting of two subtests (A and B, respectively). It is frequently used as a neuropsychological test and has been shown to be a good predictor of cognitive domains such as visual search, scanning, processing speed, mental flexibility, and executive functions (Tombaugh, 2004). It is sensitive to several neurological conditions, such as stroke and Alzheimer’s disease (Tamez et al., 2011; Shindo et al., 2013; Terada et al., 2013). TMT A and B have also been found useful in assessing driving ability (Marshall et al., 2007; Roy and Molnar, 2013).

TMT A consists of 25 numbers (1–25) that are spread over an A4 sheet, and the participant is asked to draw lines between the numbers in ascending order as quickly as possible. This subtest demands psychomotor speed and visual perception skills. TMT B is more complex and puts higher demands on executive abilities that require shifting attention and mental flexibility (Classen et al., 2013). The subtest is also done on an A4 sheet, this time with numbers (1–13) and letters (A–M) on the sheet. The participant is to alternate between the numbers and letters in ascending order, which demands higher levels of mental flexibility and executive functions. This is also to be done as quickly as possible.

Results for each subtest are scored as the number of seconds it took to correctly complete the test. Because TMT A and B are frequently used to assess some of the cognitive domains necessary for driving, and because of their use in previous studies of driving simulators (Urlings et al., 2018; Samuelsson and Wressle, 2021), they were chosen for this study.

#### 2.2.3 Useful field of view test

The useful field of view is described as the area from where an individual can detect visually presented information without having to turn the head or the eyes (Ball et al., 1988). This capacity is well known to be associated with driving ability (Wood and Owsley, 2014). The useful field of view is affected by several factors, such as visual functions, processing speed, divided attention and selective-attention skills (Ball et al., 1990; Owsley et al., 1995). The Useful field of view test (UFOV) is a computer-based test consisting of three subtests, with increasing cognitive complexity (Wood and Owsley, 2014). The subtests are aimed at assessing visual processing speed, divided attention and selective attention skills (Marshall et al., 2007; Urlings et al., 2018). The UFOV test has been found to be a significant predictor of driving outcome measures, specifically crash risk and driving ability (Wood and Owsley, 2014). Individuals, specifically older drivers, with low results on the test have a 2.2 times higher likelihood to be involved in a car crash over the subsequent three years (Owsley et al., 1998). Hence UFOV is recommended for, and frequently used in, the assessment of driving ability (Marshall et al., 2007). During the test situation the participant is seated in front of a computer screen. The task is to detect, identify and point out briefly presented stimuli on the screen. Before each subtest the participant is allowed one practice run, to ensure understanding.

1. In the first subtest, which measures speed of visual processing, the participant must identify a target, centrally located on the screen (either a silhouette of a car or a truck). The subtest measures central visual field and visual processing speed.
2. Subtest two aims to measure divided attention. The participant is to identify which vehicle is shown centrally on the screen, but at the same time pay attention to a peripheral object (a silhouette of a car) located somewhere on the screen.
3. Subtest three aims to measure selective attention. The task is laid out in the same way as subtest two, but the screen is now filled with distractors in the form of triangles across the entire screen.

Results on the test are generated in the form of mean values, in milliseconds, for the three subtests. Based on a composite of the three subtests the participant is placed in a category (1–5) for predicting crash risk (Classen et al., 2013).

### 2.3 Procedure

The testing was carried out by the first and last author, following the same protocol for administering the tests and all tests were done in one session and the same order for each participant. Participants were first tested with TMT A and B, followed by UFOV 1–3, and last with the simulator. Total time per participant was approximately 60 min. Each participant was awarded a cinema ticket.

### 2.4 Statistical analysis

The collected data was compiled and analyzed in the statistics programs Jamovi 2.2.5 and IBM SPSS Statistics 29.0. Initial data analysis was conducted to obtain and evaluate descriptive data. Both parametric and non-parametric methods were used, depending on the nature of the data regarding normal distribution.

Cronbach’s alpha (α) was used to examine the internal consistency of the variables included in the three different subtests of the simulator, that is, how well the variables within the simulator measures the same aspects of cognitive ability. Cronbach’s alpha greater than 0.90 was considered excellent; 0.70–0.90 as good; 0.60–0.70 as acceptable; 0.50–0.60 as poor, and below 0.50 as negligible (cf. Kline, 2013).

To examine how well the results on the simulator tool compared against the already validated cognitive tests (i.e., TMT A and B; UFOV), that is, the simulator test’s concurrent validity, a correlation analysis was conducted. Since the assumption of normality was not met, Spearman rank correlation coefficient (*r*_*s*_) was used. A correlation coefficient of *r*_*s*_ > 0.90 was considered as very high; *r*_*s*_ > 0.70 as high; *r*_*s*_ > 0.50 as moderate; *r*_*s*_ > 0.30 as low and *r*_*s*_ < 0.30 as negligible (cf. Mukaka, 2012). Considering the risk of making type I errors when performing multiple comparisons, the level of significance considered acceptable was set at *p* < 0.001. Hence, all correlations reported are significant at *p* < 0.001.

Multiple regression analysis was conducted to examine which variables might predict results on the reaction tests and lane positioning tests (i.e., crossing the Centerline; driving offroad).

The sample was divided into two groups for comparison of age-related differences. One group consisted of participants 65 years and older (*n* = 23) and the other group of participants younger than 65 years (*n* = 44), see Table 1 for demographic details.

Since the assumptions of normality of the sample was not met, a non-parametric Mann-Whitney *U*-test was conducted to compare the rank sums of the two groups, with *p* < 0.05 considered significant.

Correlations between age and results on the simulator tool were used to further examine the relationship between age and the results from the simulator test.

## 3 Results

### 3.1 Internal consistency

When looking at all 26 variables combined the simulator tool showed good internal consistency, with α = 0.83.

### 3.2 Correlations with other cognitive tests

Regarding the reaction time tests, the analysis showed that reaction time in all three subtests had moderate correlation with UFOV 3. All but two, Reaction Left 1 (*r*_*s*_ = 0.46) and Double Reaction 2 (*r*_*s*_ = 0.48), was moderately correlated also with TMT A. Further, reaction time on subtest 3 was moderately correlated with UFOV 2. In subtests 1 and 2, low correlations were found with UFOV 2. Low correlations were also found between the reaction tests and UFOV 1 in all but one variable (Double Reaction 3, *r*_*s*_ = 0.53). Lane positioning tests (Position-Edge line/Offroad and Position-Centerline) showed low to moderate correlations with UFOV 3 in all subtests. A few correlations at the moderate level were found for the variables measuring incorrect reactions and misses and all subtests of the UFOV test. However, there was a lack of consistent or predictable alignment between the errors made in the driving simulator and the performance on the UFOV subtests, hence these showed no clear congruency (see Table 2).

**TABLE 2:** Spearman rank correlations between the simulator subtests and the cognitive tests UFOV and TMT A and B, respectively.

Test	UFOV1	UFOV2	UFOV3	TMTA	TMTB	Age
**Simulator subtest 1**						
React total	0.44	0.48	0.57*	0.52*	0.36	0.76*
React left	0.44	0.50	0.56*	0.46	0.36	0.79*
React right	0.40	0.44	0.53*	0.54*	0.36	0.70*
Incorrect left	0.43	0.22	0.18	0.31	0.06	0.11
Incorrect right	0.17	0.13	0.24	0.26	0.24	0.25
Missed left	0.58*	0.48	0.48	0.28	0.15	0.59*
Missed right	0.27	0.33	0.26	0.38	0.29	0.49
Position–Centerline	0.27	0.11	0.40	0.22	0.10	0.59*
Position–Edge line/offroad	0.33	0.32	0.52*	0.35	0.29	0.68*
**Simulator subtest 2**						
Double react	0.45	0.49	0.58*	0.48	0.31	0.76*
Double incorrect	0.05	0.04	0.12	0.06	-0.05	-0.08
Double missed	0.56*	0.43	0.26	0.30	0.29	0.30
Position–Centerline	0.30	0.15	0.45	0.27	0.20	0.59*
Position–Edge line/offroad	0.27	0.31	0.51*	0.31	0.20	0.65*
**Simulator subtest 3**						
React total	0.45	0.57*	0.60*	0.54*	0.38	0.76*
React left	0.40	0.55*	0.62*	0.52*	0.39	0.74*
React right	0.46	0.58*	0.57*	0.52*	0.38	0.73*
Double react	0.53*	0.61*	0.59*	0.52*	0.38	0.71*
Incorrect left	0.18	0.11	0.05	0.12	0.01	0.06
Incorrect right	0.34	0.30	0.33	0.35	0.28	0.31
Missed left	0.30	0.27	0.23	0.27	0.18	0.31
Missed right	0.51*	0.26	0.17	0.20	0.09	0.27
Double incorrect	0.07	0.23	0.32	0.27	0.19	0.41
Double missed	0.45	0.22	0.30	0.24	0.08	0.24
Position–Centerline	0.31	0.21	0.49	0.29	0.17	0.67*
Position–Edge line/offroad	0.27	0.20	0.38	0.22	0.15	0.53*

*r_s_ > 0.50 and *p* < 0.001. UFOV, Useful Field of View Test; TMT, Trail Making Test.

In order to further examine the relationship between the simulator tool and the cognitive tests multiple regression analysis was used. When comparing results of the different subtest of the simulator test the highest mean values were found on the reaction time and lane positioning tests on subtest 3 (see Table 3). Hence, these were considered to be the most difficult tests, why Double React 3, Reaction All 3, Centerline 3 and Offroad 3 were used as dependent variables in the regression analysis.

**TABLE 3:** Means, confidence interval (CI) and standard deviation (*SD*) on the simulator test, TMT A and B and UFOV.

Test	Means (*N* = 67)	95% CI	*SD*
UFOV subtest 1	16.19	14.44–17.93	7.31
UFOV subtest 2	33.51	23.89–43.12	40.15
UFOV subtest 3	86.45	73.54–99.37	53.94
TMT subtest A	28.63	25.52–31.73	12.96
TMT subtest B	72.01	63.83–80.19	34.18
**Simulator subtest 1**			
Reaction total	1.14	1.08–1.19	0.22
Reaction left	1.11	1.06–1.16	0.22
Reaction right	1.16	1.11–1.22	0.23
Incorrect left	0.52	0.11–0.93	1.70
Incorrect right	0.67	0.25–1.08	1.73
Missed left	0.37	0.17–0.57	0.85
Missed right	0.22	0.08–0.36	0.57
Position–Centerline	5.89	5.04–6.74	3.53
Position–Edge line/offroad	3.04	2.49–3.59	2.28
**Simulator subtest 2**			
Double reaction	1.55	1.48–1.61	0.27
Double incorrect	0.96	0.71–1.19	0.99
Double missed	0.17	−0.01–0.34	0.81
Position–Centerline	5.16	4.30–6.02	3.58
Position–Edge line/offroad	2.62	2.09–3.15	2.21
**Simulator subtest 3**			
Reaction total	1.22	1.15–1.28	0.26
Reaction left	1.20	1.13–1.27	0.28
Reaction right	1.23	1.17–1.29	0.25
Double reaction	1.56	1.49–1.63	0.27
Incorrect left	0.19	0.06–0.32	0.53
Incorrect right	0.25	0.06–0.44	0.78
Missed left	0.07	−0.02–0.17	0.40
Missed right	0.07	−0.01–0.16	0.36
Double incorrect	0.96	0.61–1.30	1.44
Double missed	0.16	0.01–0.31	0.62
Position–Centerline	7.61	6.33–8.89	5.34
Position–Edge line/offroad	4.95	4.01–5.89	3.94

UFOV, Useful Field of View Test; TMT, Trail Making Test.

Results with Double Reaction 3 as dependent variable showed a significant regression equation, *F*_(5, 61)_ = 13.1, *p* < 0.001, with an explained variance of 52% (*R*^2^ = 0.52). UFOV 3, *p* < 0.001, β = 0.002, was the only significant covariate, see Table 4.

**TABLE 4:** Multiple regression analysis with double reaction 3 as dependent variable.

Covariate	β	*t*	*p*
Intercept	1.10	13.87	<0.001
TMT A	0.005	1.82	0.08
TMT B	<0.001	0.35	0.73
UFOV 1	0.005	1.35	0.18
UFOV 2	<0.001	0.25	0.81
UFOV 3	0.002	3.69	<0.001

UFOV, Useful Field of View; TMT, Trail Making Test.

Results with Reaction All 3 as dependent variable also showed a significant regression equation, *F*_(5, 61)_ = 13.50, *p* < 0.001, with an explained variance of 52%. TMT A (*p* = 0.003, β = 0.008) and UFOV 3 (*p* < 0.001, β = 0.002) were the only significant covariates, see Table 5.

**TABLE 5:** Multiple regression with Reaction total 3 as dependent variable.

Covariate	β	*t*	*p*
Intercept	0.78	10.37	<0.001
TMT A	0.008	3.10	0.003
TMT B	<-0.001	-0.42	0.68
UFOV 1	0.003	0.87	0.39
UFOV 2	<-0.001	-0.62	0.54
UFOV 3	0.002	4.12	<0.001

UFOV, Useful Field of View; TMT, Trail Making Test.

Results with Centerline 3 as dependent variable showed a significant regression equation, *F*_(5, 61)_ = 3.94, *p* = 0.004, with an explained variance of 24% (*R*^2^ = 0,244). UFOV 3 (*p* = 0.013, β = 0.035) was the only significant covariate, see Table 6. The regression analysis with Offroad 3 as dependent variable showed no significant result.

**TABLE 6:** Multiple regression analysis with Centerline 3 as dependent variable.

Covariate	β	*t*	*p*
Intercept	1.705	0.88	0.38
TMT A	0.045	0.61	0.55
TMT B	-0.017	-0.61	0.55
UFOV 1	0.182	1.84	0.07
UFOV 2	-0.004	-0.18	0.86
UFOV 3	0.035	2.57	0.01

UFOV, Useful Field of View; TMT, Trail Making Test.

### 3.3 Differences in results based on age

There were significant differences between the two groups in all test results, except for Incorrect Left 1, Incorrect Right 1, Double Incorrect 2, Incorrect Left 3, Incorrect Right 3 and Double Missed 3 (see Table 7). Large effect sizes (*r* > 0.50) were found for all reaction, edge line/offroad and centerline variables. The reaction tests were found to consistently have effect sizes larger than *r* = 0.80. Small to medium effect sizes (*r* < 0.50) were found for all incorrect reactions and misses, except for Missed Left 1 (*r* = 0.57).

**TABLE 7:** Means (and standard deviations) on the simulator tests; *p*-values and effect sizes (*r*) for Mann-Whitney *U*-tests.

Test	<65	>65	*p*	*r*
**Simulator subtest 1**				
Reaction total	1.02 (0.14)	1.37 (0.18)	<0.001	0.92
Reaction left	1.00 (0.13)	1.33 (0.19)	<0.001	0.88
Reaction right	1.04 (0.14)	1.34 (0.19)	<0.001	0.88
Incorrect left	0.18 (0.39)	1.17 (2.78)	0.18	0.14
Incorrect right	0.32 (0.67)	1.35 (2.72)	0.09	0.20
Missed left	0.04 (0.211)	1.00 (1.21)	<0.001	0.57
Missed right	0.02 (0.15)	0.61 (0.84)	<0.001	0.41
Position–Centerline	4.61 (2.67)	8.35 (3.75)	<0.001	0.64
Position–Edge line/offroad	1.93 (1.79)	5.17 (1.49)	<0.001	0.81
**Simulator subtest 2**				
Double reaction	1.40 (0.16)	1.82 (0.22)	<0.001	0.91
Double incorrect	1.00 (0.91)	0.87 (1.14)	0.29	0.14
Double missed	0.00 (0.00)	0.52 (1.34)	0.002	0.21
Position–Centerline	3.73 (2.49)	7.91 (3.81)	<0.001	0.69
Position–Edge line/Offroad	1.64 (1.71)	4.52 (1.79)	<0.001	0.75
**Simulator subtest 3**				
Reaction total	1.09 (0.16)	1.48 (0.21)	<0.001	0.89
Reaction left	1.06 (0.18)	1.47 (0.25)	<0.001	0.84
Reaction right	1.10 (0.16)	1.49 (0.21)	<0.001	0.86
Double reaction	1.42 (0.16)	1.85 (0.23)	<0.001	0.91
Incorrect left	0.14 (0.35)	0.30 (0.77)	0.60	0.04
Incorrect right	0.09 (0.29)	0.56 (1.24)	0.11	0.14
Missed left	0.00 (0.00)	0.22 (0.68)	0.016	0.13
Missed right	0.00 (0.00)	0.22 (0.60)	0.016	0.13
Double incorrect	0.64 (0.81)	1.57 (2.09)	0.024	0.31
Double missed	0.05 (0.21)	0.39 (0.99)	0.072	0.13
Position–Centerline	5.18 (3.77)	12.27 (4.87)	<0.001	0.80
Position–Edge line/offroad	3.59 (3.63)	7.57 (3.17)	<0.001	0.62

Results on reaction tests are in milliseconds. Position–Centerline, Position–Edge line/offroad, Misses and Incorrect reactions are in number of errors made.

Results of the correlation analysis showed high correlation between age (*r*_*s*_ > 0.70) and all reaction tests and a moderate correlation (*r*_*s*_ > 0.50) between age and the lane positioning tests (Offroad and Centerline), see Table 2. Regarding incorrect reactions and misses the correlation analysis showed negligible (*r*_*s*_ < 0.30) to low (*r*_*s*_ > 0.30) correlations, except for Missed Left 1 (*r*_*s*_ = 0.59) and Missed Right 1 (*r*_*s*_ = 0.49).

## 4 Discussion

Simulator driving allows assessment of driving-related cognitive abilities in a safe, standardized, and controlled way, with higher face validity than other cognitive tests. Further, considering that many older drivers avoid difficult driving situations, indications of deficits in cognitive abilities relevant for driving might not be noticed. The simulator adds the opportunity to measure cognitive abilities in a controlled environment, hence, disentangling the effects of judgment from the cognitive abilities meant to be measured (Shanmugaratnam et al., 2010).

The subtests included in the simulator test show good internal consistency (α = 0.83). Further, the simulator test showed concurrent validity with other cognitive tests being used for assessment of cognitive abilities relevant for driving (e.g., UFOV 3 and TMT A). The simulator test was also sensitive for age-related performance, considering the significant differences being found between age groups.

The high internal consistency of the simulator test indicates that the different subtests are highly consistent with each other, which suggests that all subtests measure the same cognitive abilities, and that at least attentional abilities and processing speed are cognitive abilities involved when using the simulator. However, to determine the consistency and stability over time, test–retest reliability should be analyzed.

The significant and moderate correlations between the reaction-time subtests of the simulator, on one hand, and TMT A and UFOV 3, on the other, validate that the simulator test should measure relevant psychological properties. This is because TMT A and UFOV 3 both are established measures of cognitive abilities relevant for driving (Wood and Owsley, 2014; Urlings et al., 2018; Samuelsson and Wressle, 2021). UFOV 3 measures visual attention, especially selective attention, and processing speed, while TMT A demands psychomotor speed and visual perception skills (Tombaugh, 2004; Wood and Owsley, 2014). The correlation analysis showed moderate correlations between some of the reaction subtests and UFOV 2, measuring visual divided attention and processing speed, indicating that the simulator reaction tests also demand aspects of divided attention. The moderate correlation between the simulator reaction time and these two cognitive tests suggest that the simulator test measures similar cognitive abilities.

The fact that UFOV 3 was found to be a significant explanatory variable for Double Reaction 3 and UFOV 3 and TMT A for Reaction Total 3 further suggests that there is some overlap or relationship between these measures and the reaction time tests. However, this also indicates that other factors or variables contribute to the remaining variability not found in any of the cognitive tests being used. The simulator test also demands reaction speed, which is not a cognitive ability being measured in the UFOV tests, which might further explain this result. However, reaction-time tests are often used to assess processing speed as well, which is indeed a cognitive ability measured in the cognitive tests in this study.

The correlation and regression analyses suggest that the lane-positioning subtests are low to moderately correlated with UFOV 3. This indicates that the ability to maintain lane position may be related to visual processing and attentional abilities, which is well in line with Depestele et al. (2020). These combined results can be interpreted such that the ability to maintain lane position and react to visual stimuli may be related to visual attention and processing abilities—which is consistent with previous research linking visual attention and processing speed to driving ability (McKenna, 1998; Marshall et al., 2007; Mathias and Lucas, 2009; Owsley and McGwin, 2010).

Comparison of rank sum between the two age groups showed significantly different results on reaction time and lane positioning subtests. The correlation analysis between age and results of the simulator subtests confirmed these results, showing strong correlation between age and results on the reaction test and moderate correlation between age and lane position tests. This finding is well in line with Classen et al. (2013), where it was found that both process and reaction speed, and attention skills, decline with age. Depestele et al. (2020) found older drivers to have poorer lane position behavior, specifically regarding lane positioning, as compared to younger drivers. Further, attention skills, especially divided and selective attention, executive functions, perceptual skills and processing speed were significant predictors (Depestele et al., 2020).

The Dutch original version of the simulator was considered as a test also of simultaneous capacity (Brouwer et al., 1991). Simultaneous capacity refers to the ability to process multiple pieces of information simultaneously, such as monitoring multiple objects, tracking moving targets, or responding to multiple stimuli presented concurrently. In all subtests, it is required to both maneuver the car while at the same time keep attention on, and reacting to, the traffic signs/arrows appearing on the screen. It is reasonable to suggest that this demands simultaneous capacity, which is a cognitive aspect not measured in the other cognitive tests used in this study. While subtest 2 of the UFOV test does assesses divided attention which involves elements of simultaneous capacity, it is a broader test that evaluates various aspects of visual attention and processing speed, but not specifically targeting simultaneous capacity. Further, in subtests 2 and 3 there are aspects of the executive function of inhibition involved, since the participant is required *not* to react on arrows pointing in different directions. Also, working memory is possibly involved in subtest 3 where the participant is to keep several rules regarding how and when to react to the different arrows showing on the screen. These executive functions of working memory and inhibition are not measured in any of the cognitive test used in this study. It is possible that these aspects affected the strength of the correlations being found in the analysis. The result of the regression analysis showed that UFOV 3, and partly TMT A, were the only explanatory variables for the results on reaction time and lane positioning, which further suggests that these simulator subtests also measure other cognitive abilities. It would therefore be interesting to compare the simulator test to cognitive tests measuring these—other—cognitive abilities.

Regarding the cognitive tests used for comparisons, TMT B is the only test measuring a clear executive function, specifically mental flexibility (Classen et al., 2013). The correlation analysis showed that TMT B had the lowest correlation with the reaction time and lane positioning subtests in the simulator. Further, the correlations found were significant at *p* > 0.05 and *p* > 0.01, hence not considered acceptable in this study. This suggests that the simulator test might not capture this specific aspect of executive function.

While the results of the validation of the simulator are promising, there are some limitations that need to be taken into consideration. Regarding methodological aspects of the study, the sample being used for the study should be addressed. The sample size was quite small, which could affect the power of the results and affecting the generalizability of the study. The inclusion criteria for participation were clearly defined and excluded individuals who had suffered from stroke, dementia, or had other medical conditions or medication that can affect cognition, thereby reducing the risk of confounding variables affecting the results. However, the medical status was self-reported and no further control was done to ensure that the participants in fact were healthy. Further, other possible confounding variables were not considered. Since the sample only consisted of healthy individuals, it was not corresponding to the group for which the simulator would be used. Considering that all tests being administered are developed to identify cognitive deficits, there is a risk of ceiling or floor effects for healthy individuals. There were signs of this, especially in the UFOV tests and incorrect reactions and misses on the simulator test.

There were very few incorrect reactions and misses by the participants in the simulator test. Considering that this sample consisted of a group of healthy participants these results are not surprising. Moreover, their results on the cognitive tests were in line with age-related norm values (Selander et al., 2020). A descriptive comparison of results on TMT A and B and the UFOV test between the sample and norm values for both tests (Selander et al., 2020) showed that the participants performed within one standard deviation of the mean on all tests, except for UFOV 3 where the older group performed more than one standard deviation better than the norm. The sample could therefore in large part be considered as performing well in line with the general population. However, the simulator’s variables are thought to be of more challenge to a clinical (patient) group, however, this still needs to be examined in future fitness-to-drive studies.

A total of three participants in the older group chose to discontinue the study due to motion sickness (so called simulator illness) during the testing. Some studies have found a correlation between age and higher occurrence of simulator illness, however, these findings are not conclusive (Henriksson, 2009). Regarding cognitive conditions and the occurrence of simulator illness, it appears that this is yet to be explored. However, sensitivity to simulator illness can be caused by other conditions, such as respiratory diseases, ear infections, alcohol use or different types of medications (Henriksson, 2009). These variables were not controlled for in this study. The sharpest turns of the road in the simulator environment may be made less sharp in order to decrease the risk of simulator sickness.

Future studies are also needed concerning the ability to predict driving performance, crash risk or to compare the simulator test with on-road test results. In conclusion, the evaluated simulator test shows potential for being used in fitness-to-drive assessments. Considering the significant correlations with other cognitive tests, it appears to measure abilities relevant for driving, such as attention and processing speed. Moreover, as concentration and sustained attention are also needed while driving, the simulator test may be a useful tool. Although its good internal consistency, further studies of reliability should be made, specifically regarding test-retest reliability. Considering that the simulator would be used on individuals suffering either from a neurodegenerative disease, such as Alzheimer’s disease, or other forms of neurological conditions, further studies including patient groups are necessary to evaluate clinical validity and relevance. However, it is important to keep in mind that a stand-alone test should not be used to determine clients’ driving capacity, as the clients may be incorrectly identified as unsafe drivers (false positives) or safe drivers (false negatives).

## Data availability statement

The raw data supporting the conclusions of this article will be made available by the authors, without undue reservation.

## Ethics statement

The studies involving humans were approved by the Swedish Ethical Review Authority (Dnr 2022-04778-01). The studies were conducted in accordance with the local legislation and institutional requirements. The participants provided their written informed consent to participate in this study.

## Author contributions

MG: Conceptualization, Data curation, Writing – original draft, Formal analysis, Methodology. RJ: Supervision, Writing – original draft. BL: Supervision, Writing – original draft, Conceptualization, Methodology. HS: Conceptualization, Supervision, Writing – original draft, Data curation, Funding acquisition.
